# Jasmonate enhances cold acclimation in jojoba by promoting flavonol synthesis

**DOI:** 10.1093/hr/uhae125

**Published:** 2024-05-03

**Authors:** Lamei Zheng, Bojing Li, Genfa Zhang, Yijun Zhou, Fei Gao

**Affiliations:** Key Laboratory of Mass Spectrometry Imaging and Metabolomics (Minzu University of China), National Ethnic Affairs Commission, Beijing 100081, China; Key Laboratory of Ecology and Environment in Minority Areas (Minzu University of China), National Ethnic Affairs Commission, Beijing 100081, China; College of Life and Environmental Sciences, Minzu University of China, Beijing 100081, China; College of Life and Environmental Sciences, Minzu University of China, Beijing 100081, China; College of Life Sciences, Beijing Normal University, Beijing 100875, China; Key Laboratory of Mass Spectrometry Imaging and Metabolomics (Minzu University of China), National Ethnic Affairs Commission, Beijing 100081, China; Key Laboratory of Ecology and Environment in Minority Areas (Minzu University of China), National Ethnic Affairs Commission, Beijing 100081, China; College of Life and Environmental Sciences, Minzu University of China, Beijing 100081, China; Key Laboratory of Mass Spectrometry Imaging and Metabolomics (Minzu University of China), National Ethnic Affairs Commission, Beijing 100081, China; Key Laboratory of Ecology and Environment in Minority Areas (Minzu University of China), National Ethnic Affairs Commission, Beijing 100081, China; College of Life and Environmental Sciences, Minzu University of China, Beijing 100081, China

## Abstract

Jojoba is an industrial oil crop planted in tropical arid areas, and its low-temperature sensitivity prevents its introduction into temperate areas. Studying the molecular mechanisms associated with cold acclimation in jojoba is advantageous for developing breeds with enhanced cold tolerance. In this study, metabolomic analysis revealed that various flavonols accumulate in jojoba during cold acclimation. Time-course transcriptomic analysis and weighted correlation network analysis (WGCNA) demonstrated that flavonol biosynthesis and jasmonates (JAs) signaling pathways played crucial roles in cold acclimation. Combining the biochemical and genetic analyses showed that ScMYB12 directly activated *flavonol synthase* gene (*ScFLS*). The interaction between ScMYB12 and transparent testa 8 (ScTT8) promoted the expression of *ScFLS*, but the negative regulator ScJAZ13 in the JA signaling pathway interacted with ScTT8 to attenuate the transcriptional activity of the ScTT8 and ScMYB12 complex, leading to the downregulation of *ScFLS*. Cold acclimation stimulated the production of JA in jojoba leaves, promoted the degradation of ScJAZ13, and activated the transcriptional activity of ScTT8 and ScMYB12 complexes, leading to the accumulation of flavonols. Our findings reveal the molecular mechanism of JA-mediated flavonol biosynthesis during cold acclimation in jojoba and highlight the JA pathway as a promising means for enhancing cold tolerance in breeding efforts.

## Introduction

*Simmondsia chinensis,* commonly known as jojoba, is a dioecious shrub belonging to the family Simmondsiaceae within the genus Simmondsia [1]. It is one of the three most important non-edible oils (*Jatropha curcas* and *Camelina sativa*) [2]. Jojoba originates in the Sonoran Desert in southwestern North America and exhibits strong resistance to high temperatures and drought stress. Jojoba oil is widely used in cosmetics, pharmaceuticals, and industrial lubricants owing to its exceptional resistance to high temperatures and pressures [3, 4]. Jojoba oil exhibits physical and chemical properties similar to those of sperm whale oil; thus, it is a suitable substitute, especially because of the prohibition of sperm whale oil. Studies have indicated that jojoba cultivation can help prevent land desertification [5], highlighting its substantial ecological value as a desert shrub.

Low temperatures significantly influence the growth, development, and geographical distribution of plants, resulting in adverse effects on crop quality and yield [6]. Plants underwent various physiological and biochemical changes in response to low-temperature stress-induced damage, including changes in enzyme activities, synthesis of plant hormones, and maintenance of ROS balance [7]. Jojoba exhibits tolerance to high temperatures and drought and is susceptible to low temperatures. Temperatures below 0°C inhibited the growth and development of jojoba [8]. The sensitivity of jojoba to low temperatures significantly impedes its cultivation in temperate and arid regions. Therefore, investigating methods to enhance the cold tolerance of jojoba is crucial. Notably, cold acclimation (i.e., exposure to a prolonged period of low non-lethal temperatures) is an effective method for improving the cold tolerance of most plants [9].

Cold acclimation involves a complex regulatory network encompassing a vast array of genes. Several cold-responsive genes, including transcription factors (TFs) and functional genes, have been identified in various plants [10]. CBF TFs played significant roles in the cold acclimation of plants. CBFs interacted with CRT/DREs in the promoter regions of various cold-responsive genes, activating their expression [11, 12]. One of the outcomes of complex gene expression regulation was that changes in metabolites were crucial for the cold acclimation of plants. Studies have shown that many metabolic pathways, such as the biosynthesis of flavonoids and sugars, were triggered during cold acclimation, leading to changes in the contents of related metabolites, which partly contributed to increased plant cold tolerance [13–15]. Studies have also shown that plant hormones played key roles in regulating the responses to low temperatures. Additionally, cold stress induced the accumulation of various plant hormones, such as abscisic acid (ABA) and JAs [16]. Omics analysis has been used to reveal the regulatory networks of plant stress responses [17]. Transcriptomic and metabolomic analyses have been employed to identify gene regulatory networks associated with cold stress responses in various plants, including wheat [18], tobacco [19], and peanut [20]. Previous research demonstrated that cold acclimation improved the cold tolerance of jojoba [21]. Nevertheless, the molecular mechanisms underlying cold acclimation remain unclear.

Flavonol is a polyphenolic compound synthesized through the flavonoid biosynthetic pathway and possesses various biological functions, including anti-ultraviolet [22], anti-insect [23], antioxidant [24, 25], and anti-abiotic stress [26]. Flavonol biosynthesis is a segment of the flavonoid metabolic pathway, and the enzymes in this pathway contain chalcone synthase (CHS), chalcone isomerase (CHI), and flavonol synthase (FLS), which catalyze chalcone to produce the intermediate naringenin and then synthesize flavonol from dihydroflavonol catalyzed by FLS [27]. In most plants, the transcriptional regulation of functional genes associated with flavonol biosynthesis is governed by MYB TFs or the MYB/bHLH/WD repeat (MBW) complex [28, 29]. *AtMYB11*, *AtMYB12*, and *AtMYB111* have been found to independently activate the expression of flavonol synthesis-related genes such as *CHS*, *CHI*, and *FLS* [30–32].

JAs, comprising jasmonic acid and its oxylipin derivatives, are crucial oxylipins in plants. They played significant roles in regulating plant growth, development, and stress response [33–35]. The components of the JA signaling pathway have been identified, including the SCF^COI1^ complex, JAZs, and MYC2 TFs [36, 37]. The JA-ZIM-domain (JAZ) proteins serve as substrates for the SCF^COI1^ complex and act as negative regulators by inhibiting multiple transcriptional regulators [38]. Upon sensing JA signals, COI1 recruits JAZs for ubiquitination and subsequent degradation to activate downstream signaling cascades that initiate the production of defense compounds against biotic and abiotic stresses [39]. Studies have shown that JA positively regulated plant cold tolerance. Cold stress induced the expression of genes involved in JA biosynthesis, increasing JA content [40, 41], and JA addition significantly improved the cold tolerance of orange [42], banana [43], and tomato [44]. Molecular studies found that JA enhanced cold tolerance in *Arabidopsis* by activating the ICE-CBF module [45]. These results demonstrated the importance of JA in the response of plants to cold stress. The regulatory mechanism of JA signaling pathway in jojoba response to cold acclimation has not been studied yet.

Jojoba genome sequencing has provided relevant data for further molecular research [46, 47]. Metabolomic and transcriptomic analyses showed that cold acclimation activated the biosynthesis of flavonol and JA. By combining biochemical and genetic analyses, we found that ScMYB12 directly activated the expression of the *ScFLS* gene; the interaction between ScMYB12 and ScTT8 promoted the expression of *ScFLS*, but the negative regulator ScJAZ13 interacted with ScTT8 to attenuate the transcriptional activity of the ScTT8 and ScMYB12 complex, leading to downregulation of *ScFLS* gene; and cold acclimation-induced JA promoted the transcriptional activity of the ScTT8/ScMYB12 complex through the degradation of ScJAZ13, further promoting the accumulation of flavonol in jojoba.

## Results

### Accumulation of flavonoids in jojoba after cold acclimation

Studies have shown that cold acclimation enhanced the cold tolerance of jojoba [21]. In this study, phenotypic analysis revealed no significant changes in jojoba after 0, 6, 24, 48, and 72 h and 1 week of cold acclimation at 15°C/10°C (day/night) (Fig. S1, see online supplementary material). We speculated that the increased cold tolerance in jojoba may be associated with the accumulation of certain metabolites. To reveal the alterations in metabolite pre-cold and post-cold acclimation in jojoba, we used widely targeted metabolomics to assess the variances in metabolites between the control group (28°C/26°C day/night, 1 week, CK) and cold acclimation group (15°C/10°C, day/night, 1 week, CA). PCA and the heat map of all metabolites revealed a significant separation in the metabolic profiles of CK and CA, indicating that substantial change in metabolites occurred after cold acclimation in jojoba leaves (Fig. S2a and b, see online supplementary material). A total of 918 metabolites were detected in jojoba leaves (Table S1, see online supplementary material) and were categorized into 12 groups. The most enriched category was flavonoids (261 metabolites), followed by phenolic acids (134 metabolites), lipids (125 metabolites), and amino acids and their derivatives (75 metabolites) (Fig. S2c, see online supplementary material). A replacement test of the PLS-DA model revealed a significant difference in the abundances of some metabolites between CK and CA (Fig. S2d, see online supplementary material). VIP analysis revealed that the metabolites with the highest VIP values were flavonoids, including anthocyanins, flavonols, and flavones (Table S2, see online supplementary material).

**Figure 1:**
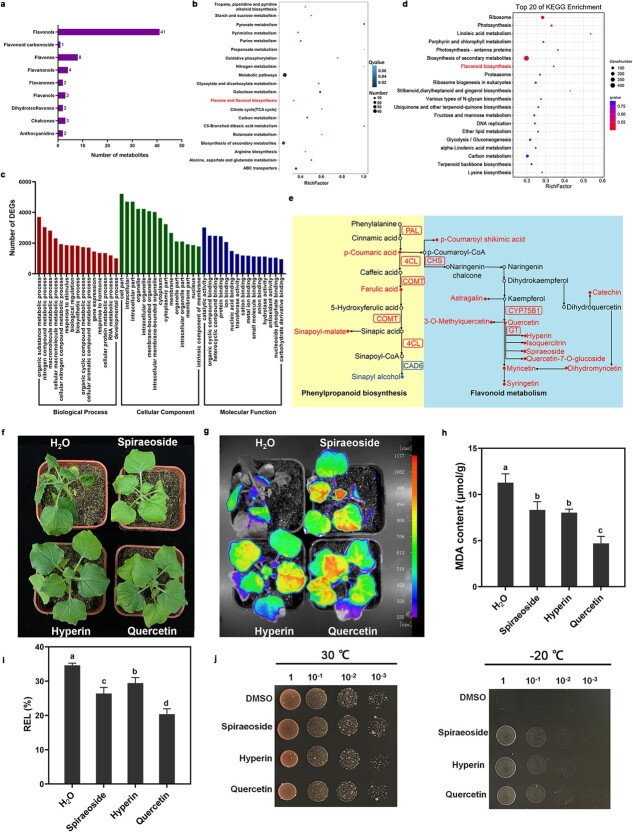
Cold acclimation stimulated flavonoid production in jojoba. **a** Classification of the flavonoids in all DAMs. **b** KEGG analysis of the DAM. **c**, **d** GO and KEGG analysis of all DEGs. **e** Metabolites transformation and the corresponding enzyme gene changes in flavonoid metabolism pathways under cold acclimation. The dots and rectangle, respectively, indicated metabolites and the genes encoding the corresponding enzymes. Red and blue represented the increase and decrease of metabolites/genes after cold acclimation, respectively. **f** The morphology changes of the treatment group (spiraeoside, hyperin, and quercetin) and the control group (H_2_O) after cold stress. **g** The delayed fluorescence imaging of the treatment groups and the control group. **h** MDA content of control group and treatment group. **i** REL measurements. Statistical analysis of the data was conducted using Duncan’s multiple range test. **j** Exogenous addition of spiraeoside, hyperin, and quercetin enhanced the cold tolerance of yeast strain W303-1A.

By comparing the metabolite chromatograms between CK and CA, 207 differentially accumulated metabolites (DAMs) were identified, with 155 showing upregulation and 52 downregulation (Table S3, Fig. S2e, see online supplementary material). The DAMs were divided into 11 categories, including 68 flavonoids, 25 lipids and phenolic acids, and 19 nucleotides and their derivatives. Notably, most metabolites in the flavonoid category showed increased abundance after cold acclimation. The flavonoids were further divided into nine subcategories: flavonol, flavone, dihydroflavonol, chalcone, flavanol, dihydroflavone, dihydroisoflavone, anthocyanin, and flavone glycoside, with flavonol accounting for the largest proportion (Fig. 1a). In the flavonol category, certain metabolites such as quercetin and spiraeoside showed noticeable accumulation after cold acclimation. KEGG enrichment analysis of the DAMs showed that the top three most enriched pathways were galactose metabolism; flavonoid and flavonol biosynthesis; and alanine, aspartate, and glutamate metabolism (Fig. 1b). These results indicated that cold acclimation induced flavonoid accumulation in jojoba leaves.

### Time-course transcriptome analysis revealed biological processes and metabolic pathways associated with cold acclimation in jojoba

Time-course transcriptomic analysis was conducted to explore the dynamic changes in gene expression during cold acclimation in jojoba. The PCA and correlation analyses showed good reproducibility within the same group (Fig. S3a and b, see online supplementary material). We used 0 h as a control, and 406 (239 upregulated, 167 downregulated), 2723 (1864 upregulated, 859 downregulated), 5112 (4226 upregulated, 886 downregulated), 1767 (698 upregulated, 1069 downregulated), and 5936 (2957 upregulated, 2979 downregulated) differentially expressed genes (DEGs) were identified at 6, 24, 48, and 72 h and 1 week, respectively (Fig. S3c, see online supplementary material). The Venn diagram showed a low number of overlapping genes among the DEGs in different groups, indicating that distinct biological events may occur at different stages of cold acclimation in jojoba (Fig. S3d, see online supplementary material). The results of the qRT-PCR analysis were in substantial agreement with the transcriptome sequencing results, indicating the credibility of the transcriptome sequencing outcomes (Fig. S3e, see online supplementary material).

GO enrichment analysis of all DEGs (identified as DEG at least at one time point) revealed the enrichment of terms associated with stress responses, such as response to stimuli, response to hormones, and antioxidant activity (Fig. 1c). KEGG analysis indicated that photosynthesis, proteasomes, porphyrin and chlorophyll metabolism, N-glycan biosynthesis, and flavonoid biosynthesis were the top five most enriched metabolic pathways (Fig. 1d).

### Combined analysis of transcriptome and metabolome revealed the involvement of the flavonoid synthesis pathway in cold acclimation

Combined analysis of the transcriptome and metabolome identified 47 co-enriched metabolic pathways (Fig. S4a, see online supplementary material). These pathways were divided into three categories: amino acid metabolism (e.g., amino acid biosynthesis and alanine, aspartate, and glutamate metabolism), sugar metabolism (e.g., galactose metabolism and starch and sucrose metabolism), and flavonoid metabolism (e.g., flavonoid and flavonol biosynthesis and phenylalanine biosynthesis) (Fig. S4b, see online supplementary material). These co-enriched metabolic pathways might play crucial roles in cold acclimation in jojoba.

Flavonoid compounds constituted the largest proportion of DAMs, with most flavonoid contents increasing after cold acclimation. To further analyse the potential gene regulation mechanism in the flavonoid metabolic pathway after cold acclimation, the differentially accumulated flavonoids and the corresponding key enzyme-coding genes were analysed jointly. Within the phenylpropanoid biosynthesis pathway, the levels of three metabolites, including p-coumaric acid and ferulic acid, were elevated after cold acclimation. This trend was in line with the upregulation of genes encoding crucial enzymes such as *PAL* and *4CL*. Within the flavonoid biosynthesis pathway, the contents of 10 flavonols, including spiraeoside, hyperin, and quercetin, increased. Most of the related enzyme-coding genes were also upregulated, including *CHS* and *glucoside transferase* (*GT*) (Fig. 1e; Table S4, see online supplementary material). These results suggested that an increase in flavonoid biosynthesis was crucial for the cold acclimation of jojoba.

The combined metabolome and transcriptome analyses showed that flavonols levels in jojoba increased significantly after cold acclimation. To evaluate the influence of flavonol accumulation on the cold resistance of jojoba, we investigated the effects of spraying tobacco seedlings with 1 mM spiraeoside, hyperin, and quercetin to understand their impact on tobacco cold tolerance. The control group displayed noticeable wilting, and the tobacco leaves treated with flavonols showed slight wilting after cold treatment (Fig. 1f), indicating that the exogenous application of flavonols improved the cold tolerance of tobacco. The delayed fluorescence of tobacco leaves treated with flavonols was substantially greater than that of the control group, indicating that the exogenous application of flavonols reduced the damage to the photosynthetic apparatus caused by cold stress (Fig. 1g). Malondialdehyde (MDA) content and relative electrolytic leakage (REL) measurements also indicated that the exogenous application of flavonols reduced cell membrane damage caused by cold stress (Fig. 1h and i). After the freezing stress treatment, yeast strain W303-1A exhibited a higher growth rate in the medium supplemented with spiraeoside, hyperin, and quercetin (Fig. 1j). These results indicated that the addition of spiraeoside, hyperin, and quercetin enhanced the cold tolerance of tobacco seedlings and yeast cells.

### Identification of key modules and genes regulating cold acclimation of jojoba by WGCNA analysis

To identify the regulatory modules that played key roles in the cold acclimation process of jojoba, we conducted WGCNA for all DEGs. All DEGs were divided into 18 modules with a minimum height of 0.1992 (Fig. S5a, see online supplementary material). The number of genes in each module varied substantially, with 5098 and 30 genes in the largest and smallest modules, respectively. Correlation analysis between these modules and the different time points indicated that the genes in the three modules of MEblack, MEsalmon, and MEdarkorange showed similar expression patterns during cold acclimation, which were positively correlated at 0, 6, 24, and 48 h, and the five modules of MEdarkgreen, MEsteelblue, MEdarkred, MEgreen, and MEroyalblue were positively correlated at 24 and 48 h, and negatively correlated at 0, 6, and 72 h. MEdarkgreen, MEgreen, MEbrown, MEmagenta, MEpaleturquoise, and MEblack were positively correlated at 1 week. Notably, the MEbrown module exhibited the highest correlation at 1 week (r = 0.98) and was negatively correlated with the other time points (Fig. S5b, see online supplementary material), indicating that this module may be involved in the response to the late stages of cold acclimation in jojobas.

**Figure 2:**
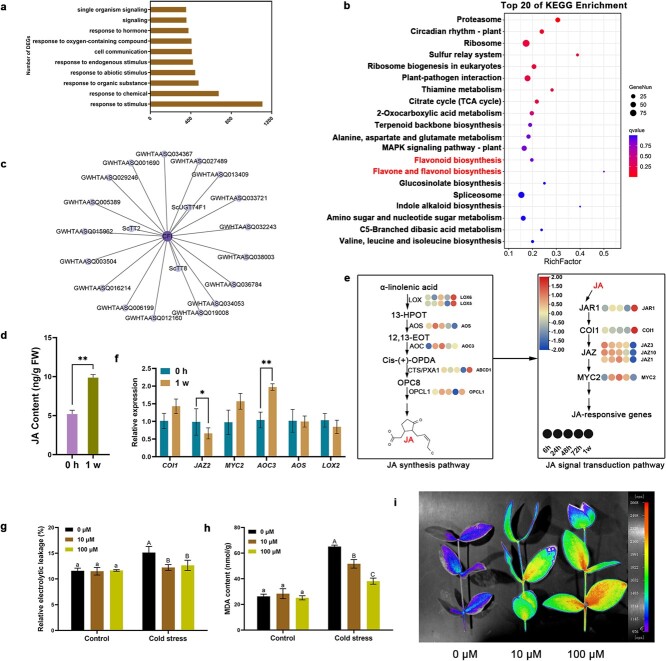
Analysis of WGCNA and synthesis and signal transduction pathways of JA. **a** Top 10 GO terms of biological process in GO enrichment analysis in the MEbrown module. **b** KEGG analysis of MEbrown module. **c** Co-expression network analysis of the MEbrown module. The gene in the middle denoted the hub gene. **d** The alterations in JA content in jojoba leaves pre- and post- cold acclimation. **e** Trend analysis of gene expression levels in JA signaling pathway. The heatmap was generated using the log2FC values of the corresponding genes. **f** The qRT-PCR analysis of crucial genes in synthesis and signal transduction pathways of JA under cold acclimation in jojoba. Each error bar showed the mean ± SD. All data were compared by an ANOVA followed by a Fisher’s least significant difference (LSD) test. ^*^P < 0.05, ^**^P < 0.01. **g** MDA content measurement of control and MeJA treatment group. **h** REL analysis of control and MeJA treatment group. **i** Delayed fluorescence imaging of control and MeJA treatment group. Lowercase letters indicate the significance analysis of different groups in the control group, and uppercase letters denote the significance analysis of different groups in the stressed group. Multiple comparisons were performed using the R package (Duncan’s multiple range test, *P* < 0.05).

To clarify the potential biological functions of the genes within the MEbrown module, we conducted comprehensive GO and KEGG analyses for all genes in the module. The results indicated significant enrichment of some biological processes and metabolic pathways associated with hormone signaling and flavonoid biosynthesis, including responses to hormones; responses to jasmonic acid; and the biosynthesis of flavonoids, flavones, and flavonols (Fig. 2a and b;  Table S5, see online supplementary material). To further investigate the genes regulating flavonoid synthesis in the MEbrown module, the core genes in the module were screened using module membership (MM) > 0.8 and gene significance (GS) > 0.3 as the criteria. The results showed that the key enzyme chalcone flavanone isomerase encoding gene *CFI* and two bHLH TFs (*ScTT2* and *ScTT8*) regulating flavonoid metabolism were the hub genes in the network (Fig. 2c).

### JA signaling pathway was crucial for cold acclimation of jojoba

Transcriptomic and WGCNA analyses indicated that hormone signaling pathways, particularly the JA signaling pathway, probably have significant regulatory functions in the cold acclimation of jojoba. LC–MS analysis showed a 90% increase in JA content in the leaves after cold acclimation (Fig. 2d), suggesting that cold acclimation induced JA production in jojoba. The changing trends of metabolites and corresponding genes in JA biosynthesis and signal transduction pathways were analysed based on transcriptomic and metabolomic data. In general, these metabolites and their corresponding genes in the JA signaling pathway were upregulated after cold acclimation. Some of these genes showed a gradual upward trend, such as *LOX*, *CTS/PXA1*, and *OPCL1*. Some genes exhibited an initial increase followed by a subsequent decrease, including *AOS* and *AOC*. The JA receptor gene *COI1* in the JA signal transduction pathway was significantly upregulated, and multiple inhibitor JAZ proteins were downregulated (e.g., *JAZ1* and *JAZ2*). The expression levels of crucial MYC2 TFs, which are responsible for regulating the downstream genes of the JA signaling pathway, exhibited a trend of first increasing and then decreasing (Fig. 2e). The qRT-PCR analysis also indicated that the changing trends of some key genes were in agreement with the transcriptome analysis (Fig. 2f). These results indicated that cold acclimation activated JA synthesis and signal transduction pathways in jojoba.

**Figure 3:**
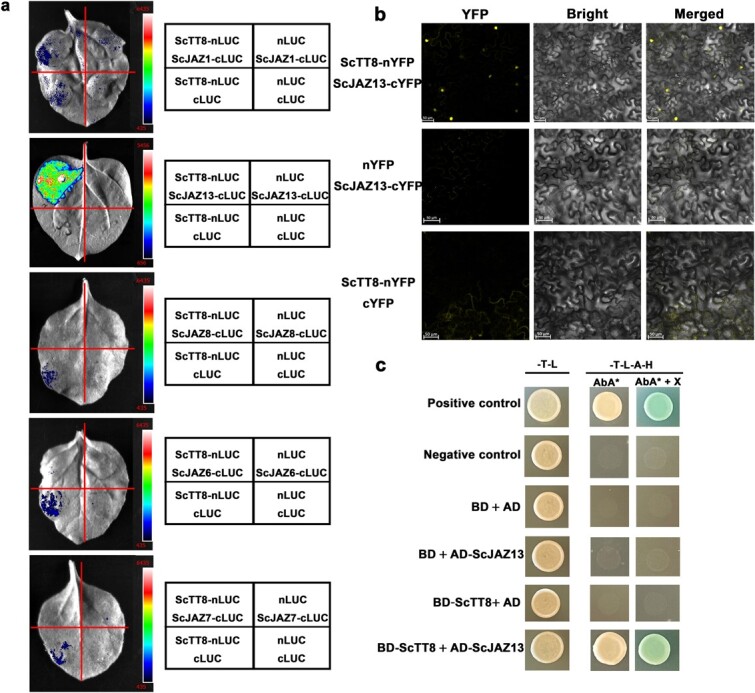
ScJAZ13 interacted with ScTT8. **a** LCA for testing the interactions of the ScTT8 with ScJAZ1, ScJAZ6, ScJAZ7, ScJAZ8, and ScJAZ13. The *ScTT8* was fused with nLUC, and these *JAZs* were fused with cLUC. The luminescence intensity was detected by Tanon-4800 Chemiluminescent Imaging System. **b** BiFC assay of ScTT8 and ScJAZ13. The *ScTT8* was fused with nYFP, and these *JAZs* were fused with cYFP. The scale bars denoted 50 μm. **c** Y2H assay for detecting the interactions of ScTT8 (fused with BD) with ScJAZ13 (fused with AD). Positive control, pGADT7-T and pGBKT7–53. Negative control, pGADT7-T and pGBKT7-lam.

**Figure 4:**
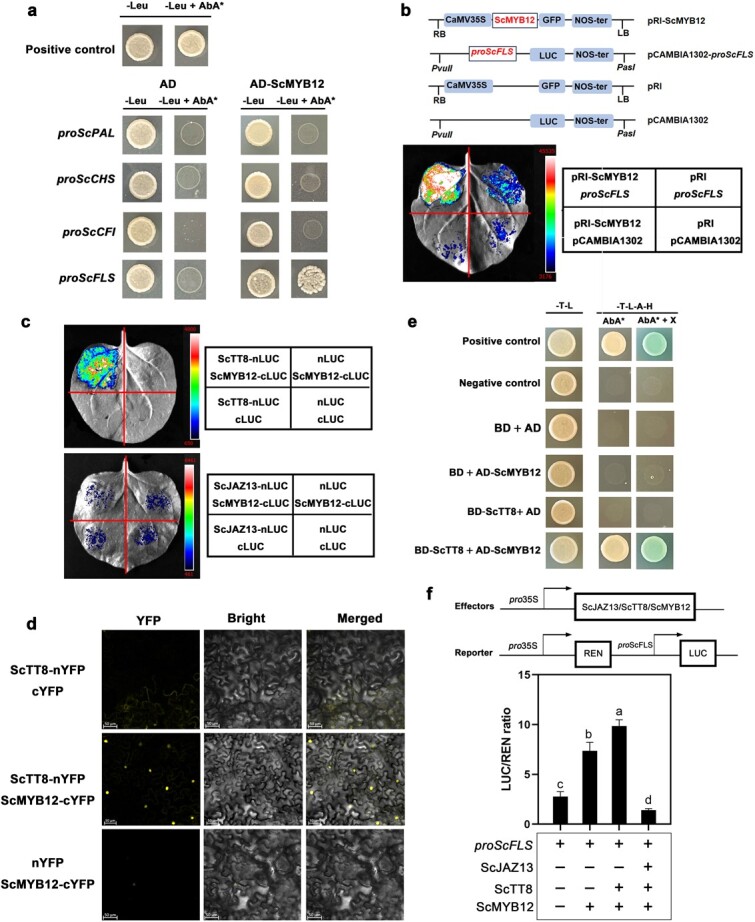
ScMYB12 interacted with ScTT8 and promoted *ScFLS* expression. **a** Y1H assay for detecting the regulatory relationship between *ScMYB12* and the promoter of *ScPAL*, *ScCHS*, *ScCFI*, and *ScFLS*. The promoter fragments of *ScPAL*, *ScCHS*, *ScCFI*, and *ScFLS* were cloned into pAbAi plasmids, and the optimal concentration of AbA to inhibit the self-activation of the promoter was screened on SD/-Ura medium. pAbAi-P53 and pGADT7–53 was used as positive controls. **b** Luciferase activity assay in tobacco leaves. The *ScMYB12* was fused with pRI-GFP, and the *proScFLS* was fused with pCAMBIA1302-LUC. The luminescence intensity was detected by Tanon-4800 Chemiluminescent Imaging System. **c** LCA of *ScMYB12* (fused with cLUC) and *ScTT8* and *ScJAZ13* (fused with nLUC) in tobacco leaves. **d** BiFC assay of ScMYB12 and ScTT8 in tobacco leaves. The *ScTT8* was fused with nYFP, and *ScMYB12* was fused with cYFP. The scale bars denoted 50 μm. **e** Y2H assay for detecting the interactions of ScTT8 (fused with BD) with ScMYB12 (fused with AD). Positive control, pGADT7-T, and pGBKT7–53. Negative control, pGADT7-T, and pGBKT7-lam. **f** Dual luciferase activity assay. The effectors *ScMYB12*, *ScTT8*, and *ScJAZ13* were ligated in pGreenII 62-SK vectors, and the reporter *proScFLS* was ligated in pGreenII 0800-LUC vectors. Statistical analysis of the data was conducted using Duncan’s multiple range test.

The impact of JA on the cold tolerance of jojoba was evaluated by measuring the physiological indexes of jojoba leaves after exogenous application of different concentrations (0, 10, and 100 μM) of methyl jasmonate (MeJA). The values of REL and MDA content in jojoba seedlings irrigated with 10 and 100 μM of MeJA solutions were significantly lower than that of jojoba seedlings irrigated with water under cold stress (Fig. 2g and h), and the delayed fluorescence imaging showed that MeJA treatment improved the tolerance of the leaf photosynthetic apparatus to damage induced via cold stress (Fig. 2i). These results suggested that the external application of MeJA alleviated the damage caused by cold stress to the cell membrane and photosynthetic apparatus.

### ScJAZ13 physically interacted with ScTT8 in jojoba

The aforementioned results showed the crucial roles of flavonol synthesis and the JA signaling pathway in the cold acclimation of jojoba. Studies have found that the key inhibitor JAZ proteins in the JA signaling pathway interacted with bHLH TFs such as TT8, GL3, and EGL3 in the MBW complex [33]. WGCNA showed that *ScTT8* was a key gene in a co-expression module that may promote flavonol biosynthesis. Thus, we hypothesized that some ScJAZs may interact with ScTT8 to regulate flavonol biosynthesis in jojoba. Thirteen *ScJAZs* were identified in jojoba (Fig. S6a, see online supplementary material). Phylogenetic analysis of JAZ proteins from jojoba and *Arabidopsis* showed that these JAZ proteins could be divided into five groups, with each group containing at least one AtJAZ protein, indicating that ScJAZ and AtJAZ proteins shared a certain degree of similarity (Fig. S6b, see online supplementary material). Studies have reported that AtTT8 interacted with AtJAZ1/2/5/6/8/9/10/11 in *Arabidopsis* [33]. Based on the phylogenetic analysis, we selected the five *JAZs* genes *ScJAZ1/6/7/8/13* to analyse whether there was an interaction between these proteins and ScTT8. The luciferase complementation assay (LCA) showed that ScJAZ13 interacted with ScTT8 and that ScJAZ1, ScJAZ6, ScJAZ7, and ScJAZ8 did not interact with ScTT8 (Fig. 3a). Next, we confirmed the interaction between ScJAZ13 and ScTT8 by using the biomolecular fluorescence complementation (BiFC) assay in tobacco (Fig. 3b). Additionally, the yeast two-hybrid (Y2H) assays revealed an interaction between ScJAZ13 and ScTT8 in yeast (Fig. 3c). In summary, these findings indicated an interaction between ScJAZ13 and ScTT8.

### ScMYB12 interacted with ScTT8 and promoted *ScFLS* expression

Studies have found that functional genes involved in flavonol biosynthesis were regulated by MYB TFs or the MBW complex [48, 49]. *MYB12* TF independently activated the expression of genes associated with flavonol biosynthesis in apple [50] and *Arabidopsis* [31]. In this study, transcriptome analysis revealed that the expression of *ScMYB12*, an ortholog of *AtMYB12* in jojoba, was significantly upregulated during cold acclimation (Fig. S7, see online supplementary material). Thus, we conducted yeast one-hybrid (Y1H) assays to determine the regulatory relationship between the *ScMYB12* and functional genes associated with flavonol biosynthesis. We found that ScMYB12 could bind to the *ScFLS* promoter but not to the *ScPAL*, *ScCHS*, or *ScCFI* promoters (Fig. 4a). The luciferase activity assay demonstrated that *ScMYB12* activated the expression of *ScFLS* (Fig. 4b).

We were interested in whether there was an interaction among ScTT8, ScJAZ13, and ScMYB12 and whether ScTT8 and ScJAZ13 could participate in regulating the expression of functional genes associated with flavonol biosynthesis through ScMYB12 in jojoba. The LCA showed that ScMYB12 interacted with ScTT8 but not with ScJAZ13 (Fig. 4c), and the BiFC and Y2H assays also confirmed the interaction between ScMYB12 and ScTT8 (Fig. 4d and e). Next, we used a dual luciferase reporter gene controlled by the *35S* and *ScFLS* promoters to investigate the effects of ScTT8, ScJAZ13, and ScMYB12 on the transcriptional activity of *ScFLS.* When *proScFLS::LUC* alone penetrated tobacco leaves, we observed a detectable level of luciferase activity, indicating that endogenous MYB TFs in tobacco activated the expression of *proScFLS::LUC*. Upon co-transfection with *ScMYB12* and *proScFLS::LUC*, there was a substantial increase in luciferase activity, suggesting that *ScMYB12* enhanced *ScFLS* expression. Additionally, when *ScMYB12*, *ScTT8*, and *proScFLS::LUC* were co-transfected, luciferase expression in tobacco leaves was further elevated, indicating that the interaction between ScMYB12 and ScTT8 enhanced the expression of *ScFLS*. Nevertheless, upon co-transfection with *ScJAZ13*, *ScTT8*, *ScMYB12*, and *proScFLS::LUC*, there was a marked decrease in luciferase expression in tobacco (Fig. 4f), indicating that ScJAZ13 suppressed the transcriptional activation of the ScTT8 and ScMYB12 complex to *ScFLS*.

### *ScMYB12* overexpression promoted expression of enzyme genes in flavanol biosynthesis pathway and conferred cold tolerance in jojoba

**Figure 5:**
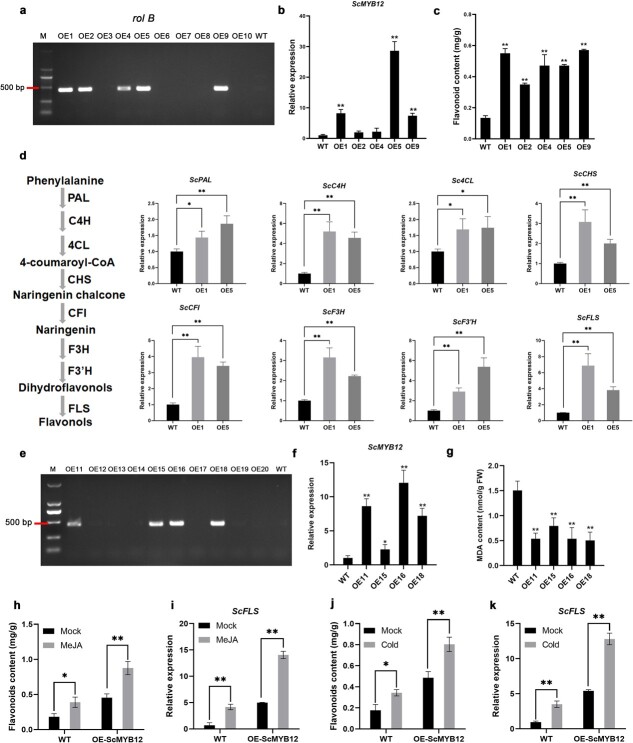
Effect of *ScMYB12* overexpression on gene expression of flavonol synthesis-related enzymes and cold stress tolerance. **a** Screening of *ScMYB12*-overexpressing hairy roots. M represented the DNA ladder DL2000, WT indicated the hairy roots of jojoba without *Agrobacterium rhizogenes* infection, and OE1-OE10 denoted different infection lines. **b** qRT-PCR analysis of hairy roots overexpressing *ScMYB12*. **c** Flavonoid content assay of hairy roots overexpressing *ScMYB12*. **d** The expression levels of enzyme genes associated with the flavonol biosynthesis were analysed in *ScMYB12*-overexpressing hairy root lines OE1 and OE5. **e** Screening of *ScMYB12*-overexpressing hairy roots. **f** qRT-PCR analysis of hairy roots overexpressing *ScMYB12*. **g** MDA content measurement. **h** Analysis of flavonoids content after MeJA treatment. **i** Analysis of *ScFLS* expression levels after MeJA treatment. **j** Analysis of flavonoids content after cold stress. **k** Analysis of *ScFLS* expression levels after cold stress. The hairy roots overexpressing *ScMYB12* were obtained by CDB delivery system, and the hairy roots growing for one month were collected for each measurement. Each experiment contained at least three independent biological replicates. Graphpad Prism 9 was used for data analysis and plotting. Each value was the mean ± SD. All data were subjected to ANOVA followed by an LSD test. ^*^P < 0.05. ^**^P < 0.01.

To assess the effects of *ScMYB12* overexpression on flavonol biosynthesis in jojoba, we constructed a genetic transformation system and obtained *ScMYB12*-overexpressed hairy roots (Fig. S8, see online supplementary material). The *rol B* gene in the Ri plasmid of *Agrobacterium rhizogenes* was detected in *OE1*, *OE2*, *OE4*, *OE5*, and *OE9* (Fig. 5a), indicating that these lines were positive hairy roots. The expression level of *ScMYB12* in these lines was significantly higher than that of the wild type (WT) (Fig. 5b). The expression levels of two key TFs closely associated with flavonoid biosynthesis, *ScTT2* and *ScTT8*, were significantly upregulated in the hairy roots overexpressing *ScMYB12* (Fig. S9, see online supplementary material). Additionally, the flavonoid content in hairy roots overexpressing *ScMYB12* was significantly increased (Fig. 5c). Expression profiling revealed a significant upregulation of enzyme genes in the flavonol synthesis pathway, such as *ScPAL*, *ScCFI*, and *ScFLS* gene (Fig. 5d). These results indicated that *ScMYB12* overexpression promoted the accumulation of flavonols in jojoba by activating the expression of enzyme genes in the flavonol biosynthetic pathway. To further analyse the effect of overexpressing *ScMYB12* on the cold tolerance of jojoba, we measured the MDA content in hairy roots after 24 h of cold stress at 4°C. The results showed the MDA content in the hairy roots overexpressing *ScMYB12* was significantly less than that of the WT hairy roots (Fig. 5e, f and g). This finding indicated that *ScMYB12* was a positive regulator of cold stress in jojoba.

The biochemical evidence above indicated that ScJAZ13 inhibited the transcriptional activity of the ScMYB12/ScTT8 complex to ScFLS. To confirm the regulatory role of the ScJAZ-MYB module in cold acclimation of jojoba, both WT and *ScMYB12* overexpressing plants were subjected to exogenous MeJA and cold stress treatments. Following treatment with 100 μM MeJA, it was found that the increase of flavonoids content in the *ScMYB12*-overexpressing hairy roots was significantly greater than that in WT, and the activation of *ScFLS* was stronger in overexpressing plants (Fig. 5h and i). Similar phenomena were observed after 24 h of cold treatment at 4°C, where the accumulation of flavonoids was significantly higher in the *ScMYB12*-overexpressing hairy roots than in WT hairy roots, and the increase in *ScFLS* expression levels was also more pronounced (Fig. 5j and k). These results indicated that ScJAZ-MYB module positively regulated the induction of flavonols by cold stress.

## Discussion

Jojoba is recognized as one of the three most crucial non-food oil plants globally and is economically crucial. In arid regions, cultivating jojoba increases incomes and helps maintain fragile ecosystems. However, the sensitivity of jojoba to low temperatures severely impedes its introduction to temperate regions. Studying gene expression networks and metabolic pathways associated with the cold tolerance of jojoba and identifying regulatory proteins that play crucial roles in cold acclimation would promote genetic breeding studies aimed at enhancing the cold resistance of jojoba.

Flavonoids, primarily anthocyanins, flavonols, and flavanols, are polyphenolic secondary metabolites in plants. In this study, metabolomic analysis showed a substantial elevation in the levels of diverse flavonoids in jojoba leaves after cold acclimation, with the highest upregulation observed in flavonol biosynthesis, particularly in quercetin, spiraeoside, and hyperoxide (Table S3, see online supplementary material). Transcriptomic analysis revealed substantial upregulation of several crucial genes associated with flavonol biosynthesis in jojoba during cold acclimation, including *PAL*, *CHS*, *CYP75B1*, and *GT* (Fig. 1e). Several abiotic stresses, such as salt [51], drought [52], and cold stress [53], have been reported to lead to the accumulation of flavonols in plants. Flavonoid synthesis was a determining factor for *Arabidopsis* to acquire freezing and cold tolerance via cold acclimation [54]. In our study, the exogenous addition of flavonols, such as quercetin, spiraeoside, and hyperoside, enhanced cold resistance in tobacco and yeast (Fig. 1f and j). Studies have reported that flavonols possessed significant antioxidant activity [55]. Several flavonols have been found in the extracts of *Bauhinia galpinii* and mulberry leaves, and the addition of these extracts significantly enhanced the ability of animal cells to scavenge free radicals [56, 57]. Therefore, we speculated that the accumulation of flavonols after cold acclimation helped jojoba manage the oxidative damage caused by cold stress.

There was a small overlap between the DEGs at different time points during the cold acclimation of jojoba (Fig. S3d, see online supplementary material), indicating that different biological events may occur at different time points. Consistent with this speculation, GO enrichment analysis identified several time point-specific and stage-specific-enriched GO biological process terms during cold acclimation. Notably, terms such as response to stimulus and response to abiotic stress showed significant enrichment at 6 h, suggesting that early biological processes in jojoba cold acclimation were primarily related to environmental stress responses. GO terms such as RNA metabolic process, gene expression, translation, protein folding, protein degradation, mitochondrial RNA modification, and chloroplast mRNA processing were mainly enriched at 24 h and 48 h, indicating that at this stage, leaf cells began to regulate the function of mitochondria and chloroplasts through gene expression and protein synthesis, rebuilding homeostasis under prolonged short-term cold acclimation. The GO terms flavonoid metabolic process and flavonoid biosynthetic process were enriched at 48 h, suggesting that the flavonoid synthesis pathway was activated at this time (Fig. 1c). Despite the occurrence of diverse biological events at different time points, some biological processes associated with hormone signaling pathways, such as plant hormone signaling and response to hormones, were enriched throughout nearly all stages of cold acclimation. This underscored the essential role of plant hormones in providing tolerance to cold stress induced by acclimation in jojoba plants. The significant positive correlation module identified by WGCNA at the late stage of cold acclimation of jojoba contained some genes associated with plant hormone signaling (Fig. 2a), further emphasizing the importance of hormones in the cold acclimation of jojoba.

JAs are a class of important signaling molecules that govern multiple biological processes related to plant growth and development and mediate plant responses to environmental stress [58–60]. Both transcriptome and WGCNA analyses indicated a crucial regulatory role for the JA signaling pathway in the cold acclimation of jojoba. The JA content of jojoba leaves increased significantly after cold acclimation. Simultaneously, the majority of genes responsible for synthesizing enzymes in the JA pathway, including *AOC* and *AOS*, along with genes encoding components of the JA signaling pathway, such as *JAR1* and *COI1*, were upregulated (Fig. 2d, e, and f). These findings indicated that JA biosynthesis was triggered by cold acclimation. In our study, jojoba seedlings treated with exogenous MeJA had lower REL and MDA levels and higher delayed fluorescence intensity under cold stress than the control group did (Fig. 2g, h, and i), suggesting that MeJA improved the cold tolerance of jojoba by reducing damage to cell membranes and chloroplasts caused by cold stress. Improved cold resistance through exogenous MeJA application has been reported in other plants, such as grapes and tomatoes [61, 62]. These results showed that JA played a crucial role in the response to cold acclimation in jojoba.

Studies have reported that JA influenced flavonol biosynthesis [63, 64]. The application of MeJA was reported to significantly elevate flavonol content in tomato seedlings [65]. Supplementing a cell culture suspension of *Polygonum multiflorum* with JA led to a notable increase in the flavonol content of the cells [66]. Therefore, we hypothesized that there may be an intrinsic connection between JA induced by cold acclimation and flavonol accumulation in jojoba. JAZ proteins played crucial roles as inhibitory factors in the JA signaling pathway and were believed to impede plant responses to JA regulation by interacting with downstream TFs [67, 68]. Some studies found that JA may affect flavonoid biosynthesis through the negative regulatory factor JAZ. In *Arabidopsis*, JAZ proteins weakened the transcriptional activity of the MBW complex by interacting with bHLH and MYB TFs, repressing JA-induced anthocyanin accumulation [33, 69]. In tea plant, CsJAZ6 interacted with the MBW components CsEGL3 and CsTTG1, and the induction of JA weakened the inhibitory effect of CsJAZ6 on CsEGL3 and CsTTG1, leading to an increase in the biosynthesis of catechins under heat stress [70]. In our study, we found 13 *ScJAZ* genes in the jojoba genome, five of which were selected for analysis of their interactions with bHLH TF—ScTT8. The LCA, Y2H, and BiFC assays indicated that ScJAZ13 interacted with ScTT8 (Fig. 3). This result implied that JA may influence flavonol biosynthesis in jojoba through ScJAZ13.

The regulatory mechanisms underlying flavonol biosynthesis have been partially elucidated [71]. *AtMYB11*, *AtMYB12*, and *AtMYB111* have been shown to directly activate the expression of *CHS*, *F3H*, and *FLS* to promote the accumulation of flavonols [30–32, 72]. In apples, MdMYB22 bound directly to the *FLS* promoter, promoting flavonol biosynthesis. *MdMYB22* overexpression in apple calli and *AtMYB12/11/111* mutants induced flavonol accumulation [50]. In pears, *PbMYB12* activation of *PbFLS* expression promoted the biosynthesis of quercetin and kaempferol glycosides [73]. In this study, *ScMYB12* expression was significantly upregulated under cold acclimation (Fig. S7, see online supplementary material). The Y1H and luciferase activity assays demonstrated that *ScMYB12* enhanced the expression of *ScFLS* by binding to its promoter (Fig. 4a and b). In jojoba hairy roots overexpressing *ScMYB12*, the flavonoid content was substantially higher than that in WT hairy roots (Fig. 5c), and the expression levels of key enzyme genes, such as *PAL*, *CFI*, and *FLS*, were significantly upregulated (Fig. 5d). The MDA content in hairy roots overexpressing *ScMYB12* decreased significantly after cold stress (Fig. 5g). These results indicated that *ScMYB12* promoted the synthesis of flavonols with antioxidant capabilities, enhancing the cold tolerance of jojoba. Furthermore, after cold stress and MeJA treatment, the increase of flavonoids content and the expression of *ScFLS* in the hairy roots overexpressing *ScMYB12* were higher compared to WT hairy roots (Fig. 5h, i, j, and k). These findings suggested that JA might positively regulate the accumulation of cold-induced flavonols through the JAZ-MYB12 module.

Studies showed that MYB TFs regulated flavonol synthesis by interacting with the bHLH members of the flavonoid-regulating complex MBW [74, 75]. In *Arabidopsis*, MYB75 enhanced the transcription of dihydroflavonol-4-reductase (DFR) by interacting with TT8 [33]. In apples, MdMYB12 activated the transcription activity of *MdFLS* through its interaction with bHLH3/bHLH33 [50]. In *Arabidopsis*, TCP3, a bHLH TF, interacted with AtMYB12 and AtMYB111, and plants overexpressing *TCP3* exhibited a significant increase in flavonol content [76]. Thus, we hypothesized that ScTT8 interacted with ScMYB12, affecting flavonol biosynthesis in jojoba. In our study, the LCA, BiFC, and Y2H assays confirmed the interaction between ScMYB12 and ScTT8 (Fig. 4c, d, and e). Co-expression of *ScTT8* with *ScMYB12* resulted in a substantial increase in the expression of *ScFLS* in tobacco leaves (Fig. 4f). This suggested that the interaction between ScMYB12 and ScTT8 enhanced flavonol accumulation by promoting the transcription of *ScFLS* in jojoba.

JAZs are a family of negative regulatory factors involved in the JA signaling pathway. Studies have reported that in some plants, such as *Arabidopsis* and tea plants, JAZ proteins interacted with bHLH or MYB TFs to negatively regulate flavonoid accumulation. In *Arabidopsis*, JAZ1 interacted with MYB75 and TT8. When *MYB75*, *TT8*, and *JAZ1* were co-expressed, the transcription levels of enzymes related to flavonoid biosynthesis, such as *DFR*, decreased, leading to a reduction in anthocyanin synthesis compared with the expression of *MYB75* alone or in combination with *TT8* [33]. In tea plants, CsJAZ6 weakened the transcription of the anthocyanin synthesis enzyme (ANS) by interacting with the CsEGL3 and CsTTG1, further repressing catechin production [70]. In this study, we found that when *ScJAZ13*, *ScTT8*, and *ScMYB12* were co-expressed, the transcription levels of *ScFLS* were significantly less than those with the expression of *ScMYB12* alone or in combination with *ScTT8* (Fig. 4f). This suggested that ScJAZ13, which acted as a negative regulatory factor, impeded the accumulation of flavonols in jojoba by suppressing the transcriptional activity of the ScMYB12 and ScTT8 complex.

Collectively, our results indicated that JA promoted the accumulation of flavonol during cold acclimation, ultimately enhancing the cold tolerance of jojoba. The mechanism by which JA regulated the cold response has been partially elucidated in other plants. In *Arabidopsis* and apples, JA regulated the CBF/DREB1 pathway-mediated cold resistance response through *AtICE1* and *MdBBX37* [45, 77]*.* In tomatoes, JA promoted ABA biosynthesis, endowing tomatoes with cold tolerance [78]. Our results indicated that JA enhanced cold tolerance by promoting the biosynthesis of flavonol with antioxidant capabilities, mediating the response to cold stress in a CBF/DREB1-independent manner. In tea, *Arabidopsis*, and apple, JA promoted flavonoid biosynthesis [33, 50, 70], implying that the mechanism by which JA promoted flavonol synthesis under cold stress observed in jojoba has a certain degree of conservation. This mechanism held a broad evolutionary advantage in maintaining such pathways to cope with environmental challenges. The discovery of the regulatory function of JAZ-MYB module in cold acclimation of jojoba suggests that plants can regulate the synthesis of flavonols by integrating environmental temperature and plant hormone signals, thereby adapting to environmental changes. In conclusion, our study reveals the molecular mechanism by which JA positively regulates jojoba’s cold tolerance through the JAZ-TT8/MYB module. At the same time, our research broadens the applicability of JAZ proteins and further indicates that the JA pathway is a valuable candidate pathway for the cold breeding and cultivation of jojoba.

## Materials and methods

### Plant materials and stress treatment

Jojoba seeds (KH-1201) were collected from Kehe Town, Sichuan Province, China. Seed sterilization and germination were performed as previously described [21]. After germination, the seedlings were transported to a greenhouse with a temperature of 28°C for cultivation. One-month-old seedlings were transferred to growth chambers (Percival Scientific, Perry, IA, USA) for cold acclimation, with conditions set at 15°C during the day and 10°C at night. Leaves from the uppermost part of each jojoba seedling were harvested at 0 (control, CK), 6, 24, 48, 72 h, and one week (cold acclimation, CA). All leaf samples were collected between 09:00 a.m. and 11:00 a.m. The gathered samples were promptly immersed in liquid nitrogen and preserved at −80°C for subsequent experiments. Leaf samples from the six time points were subjected to transcriptome analysis, and the CK and CA groups were subjected to metabolomic analysis. Detailed sampling time points are shown in Fig. S1 (see online supplementary material). Samples were collected from a minimum of three distinct jojoba seedlings, and each group was subjected to three independent biological replicates.

### Transcriptome analysis

RNA samples were obtained from jojoba leaves using the procedure of Geng *et al.* [79]. The purity, concentration, and integrity of RNA were assessed using a method described previously [21]. RNA sequencing and data pre-processing were performed at Beijing Boyun Huakang Gene Technology Co., Ltd (Beijing, China). Transcriptome data were analysed as described previously [21]. In brief, DEseq2 was employed for the detection of DEGs, with the criteria for DEG identification comprising (i) FDR < 0.05 and (ii) a fold change ≥2 or ≤ 0.5. GO and enrichment analyses were conducted as described previously [21]. Enriched metabolic pathways were screened based on Q-values. WGCNA was performed on the BMKCloud platform by using the default parameters (www.biocloud.net).

### qRT-PCR analysis

The synthesis of cDNA and the reaction for qRT-PCR were performed using a method previously described [21]. All primers were designed using Primer Premier 5.0. A comprehensive list of all primers is provided in Table S6 (see online supplementary material). *18S rRNA* (NCBI accession number: AF094562) was used as an internal control.

### Metabolomic analysis

Metabolite extraction and detection were conducted by referring to a method previously described [21]. PCA and PLS-DA of all metabolites were conducted using MetaboAnalyst 5.0 [80]. Criteria for identifying DAMs were as follows: (i) *P*. adjust (*P* value after BH adjustment) <0.05 and (ii) a fold change ≥1.5 or ≤0.67. KEGG enrichment analyses were conducted using methods described previously [21]. Enriched metabolic pathways were screened based on Q-values.

### Effects of exogenous flavonols on cold tolerance of yeast

The cold tolerance of yeast strain W303-1A was analysed using a previously described method [81]. Yeast cells were cultured in YPA medium to the exponential phase (OD_600_ between 0.6 ~ 0.8) and then resuspended in YPA medium with 0.1 mg/mL concentrations of quercetin, spiraeoside, hyperin, and DMSO, respectively. After incubating at 30°C for 30 min, yeast cells were transferred to −20°C for 36 h and cultured on YPD medium at 30°C for 2~3 d to observe the growth of colonies.

### Effects of exogenous flavonols on cold tolerance of tobacco

Forty tobacco seedlings with identical growth statuses were randomly allocated into four groups and subjected to spray treatment with 1 mM quercetin, spiraoside, hyperin, and H_2_O. Each tobacco seedling was sprayed with 10 mL of the solution. After 3 d, the tobacco seedlings were transferred to growth chambers for cold treatment at 4°C for 24 h, immediately followed by phenotypic observation and delayed fluorescence imaging. Delayed fluorescence was a noninvasive approach for assessing the impact of abiotic stress on the functionality and performance of the photosynthetic apparatus [82]. Delayed fluorescence was imaged using a NightSGADE LB 985 (Berthold, Bad Wildbad, Germany) following the previously described method [83].

### Detection of plant endogenous hormone content

The JA content in jojoba leaves after cold acclimation was assessed by Nanjing Ruiyuan Biotechnology Co., Ltd. (Nanjing, China), which used the method of Zhang *et al.* [84].

### Effects of exogenous MeJA on cold tolerance of jojoba

Sixty one-month-old jojoba seedlings with the same growth status were randomly allocated into three groups and watered with 0, 10, or 100 μM MeJA every 3 d. Six days later, half of the plants in each group were transferred to growth chambers for 24 h of cold stress at 4°C; the remaining seedlings were designated as the control. REL and MDA content were determined, and delayed fluorescence imaging of the leaves was performed. The MDA content and REL were determined using a previously described method [85].

### LCA

*ScTT8* was fused to the plasmid pCAMBIA1300-nLUC to obtain the target construct ScTT8-nLUC, and *ScJAZ1*, *ScJAZ6*, *ScJAZ7*, *ScJAZ8*, *ScJAZ13*, and *ScMYB12* were fused to the plasmid pCAMBIA1300-cLUC to obtain different constructs. The *Agrobacterium tumefaciens* GV3101 harboring the recombinant plasmid was co-transformed into tobacco leaves in different combinations. After 2~3 d of culture, the luminescence intensity was detected using a Tanon-4800 Chemiluminescent Imaging System (Tanon, China) following dark treatment for 10 min, according to a previously described method [86]. Each experiment consisted of a minimum of five independent biological replicates.

### BiFC assay

*ScTT8* was fused to plasmid pSPYNE-35S to obtain the target construct ScTT8-nYFP, and *ScJAZ13* and *ScMYB12* were fused to plasmid pSPYCE-35S to obtain the target constructs ScJAZ13-cYFP and ScMYB12-cYFP. The *A. tumefaciens* GV3101 harboring the recombinant plasmid was co-transformed into tobacco leaves in different combinations. After 2~3 d, YFP fluorescence was observed using a confocal microscope (Leica, Mannheim, Germany).

### Y2H assay

*ScTT8* was fused into the pGBKT7 (BD) plasmid to generate the target construct BD-ScTT8, and *ScJAZ13* and *ScMYB12* were fused into the pGADT7 (AD) plasmid to generate the target constructs AD-ScJAZ13 and AD-ScMYB12, respectively. Different combinations were transformed into the yeast strain Y2H-Gold (Coolaber, Beijing, China). The yeast cells were cultured at 28°C on SD/−Trp/−Leu (−T-L) plates for 48~96 h. Subsequently, the colonies were transferred to SD/−Trp/−Leu/-His/−Ade (−T-L-A-H) plates with the addition of Aureobasidin A (AbA) and X-α-gal for growth.

### Y1H assays

The promoter sequences (1500 bp upstream of the start codon) of *ScPAL*, *ScCHS*, *ScCFI*, and *ScFLS* were cloned into the pAbAi yeast-integrating vector to obtain different constructs. Different promoter constructs were transformed into yeast strain Y1H-Gold (Coolaber, Beijing, China). Yeast cells were cultured at 28°C on SD/-Ura plates. Subsequently, the optimal concentration of AbA required to inhibit the self-activation of the promoter was screened on SD/-Ura plates. The target construct, AD-ScMYB12, was obtained by inserting full-length *ScMYB12* into AD plasmids. The obtained AD-ScMYB12 was transformed into the yeast strain Y1H-Gold cells harboring different promoter fragments. The colonies were cultured on SD/−Leu plates at the optimal concentration of AbA.

### Luciferase activity assay

The *ScMYB12* and *proScFLS* sequences were inserted into pRI-GFP and pCAMBIA1302-LUC using homologous recombination to obtain the target constructs, pRI-ScMYB12 and pCAMBIA1302-proScFLS. The *A. tumefaciens* GV3101 harboring the recombinant plasmid was co-transformed into tobacco leaves in different combinations. After 2~3 d, the luciferase activity in tobacco leaves was analysed using the Tanon-4800 Chemiluminescent Imaging System (Tanon, China) with the procedure outlined by Cheng *et al.* [87].

### Dual luciferase activity assay

The *ScFLS* promoter and *ScMYB12*, *ScTT8*, and *ScJAZ13* were inserted into the pGreenII 0800-LUC and pGreenII 62-SK vectors to generate the reporter and effector constructs, respectively. The *A. tumefaciens* GV3101 harboring the recombinant plasmid was co-transformed into tobacco leaves in different combinations. After 2~3 d, luciferase activity was assessed using a dual luciferase reporter assay kit (Vazyme, Nanjing, China).

### Transformation of hairy roots of jojoba

The genetic transformation system for jojoba was constructed according to a cut-dip-budding (CDB) delivery system by using a previously described method [88]. *ScMYB12* was cloned into the pCAMBIA1305 vector to generate the *35S:ScMYB12* construct. Hairy roots of jojoba overexpressing *ScMYB12* were obtained using a CDB delivery system, and PCR amplification of *rol B* from *A. rhizogenes* was performed to confirm the success of the transgenic process [89]. The total flavonoid content in the hairy roots of different lines was determined using a plant flavonoid assay kit (Solarbio, Beijing, China). The *ScMYB12*-overexpressing plants were watered every three days with 100 μM MeJA. After six days, the flavonoid content and gene expression levels in the hairy roots were quantified.

## Acknowledgements

This study was funded by the National Natural Science Foundation of China (Grant numbers 31770363 and 31670335), and Beijing Advanced Discipline for Mass Spectrometry Imaging and Metabolomics (No. 104-01900403).

## Author contributions

F.G., Y.Z., and G.Z. conceived and designed the experiments. L.Z. and B.L performed experiments and analysed the data.

## Data availability

All raw RNA-seq data were deposited in GeneBank with the accession number PRJNA778341.

## Conflict of interest statement

The authors declare no conflicts of interest.

## Supplementary data

Supplementary data is available at *Horticulture Research* online.

## Supplementary Material

Web_Material_uhae125

## References

[1] Shah SN, Sharma BK, Moser BR. et al. Preparation and evaluation of jojoba oil methyl esters as biodiesel and as a blend component in ultra-low sulfur diesel fuel. BioEnergy Res. 2010;3:214–23

[2] Yang L, Takase M, Zhang M. et al. Potential non-edible oil feedstock for biodiesel production in Africa: a survey. Renew Sust Energ Rev. 2014;38:461–77

[3] Agarwal S, Kumari S, Mudgal A. et al. Green synthesized nanoadditives in jojoba biodiesel-diesel blends: An improvement of engine performance and emission. Renew Energy. 2020;147:1836–44

[4] Agarwal S, Arya D, Khan S. Comparative fatty acid and trace elemental analysis identified the best raw material of jojoba (*Simmondsia chinensis*) for commercial applications. Ann Agric Sci. 2018;63:37–45

[5] Ashraful AM, Masjuki HH, Kalam MA. et al. Production and comparison of fuel properties, engine performance, and emission characteristics of biodiesel from various non-edible vegetable oils: a review. Energy Convers Manag. 2014;80:202–28

[6] Zhang J, Li XM, Lin HX. et al. Crop improvement through temperature resilience. Annu Rev Plant Biol. 2019;70:753–80 31035832 10.1146/annurev-arplant-050718-100016

[7] Hincha DK, Zuther E. Introduction: plant cold acclimation and winter survival. Methods Mol Biol. 2020;2156:1–7 32607970 10.1007/978-1-0716-0660-5_1

[8] Al-Obaidi JR, Halabi MF, AlKhalifah NS. et al. A review on plant importance, biotechnological aspects, and cultivation challenges of jojoba plant. Biol Res. 2017;50:25 28838321 10.1186/s40659-017-0131-x PMC5571488

[9] Juurakko CL, diCenzo GC, Walker VK. Cold acclimation and prospects for cold-resilient crops. Plant Stress. 2021;2:100028

[10] Kidokoro S, Shinozaki K, Yamaguchi-Shinozaki K. Transcriptional regulatory network of plant cold-stress responses. Trends Plant Sci. 2022;27:922–35 35210165 10.1016/j.tplants.2022.01.008

[11] Hwarari D, Guan Y, Ahmad B. et al. ICE-CBF-COR signaling cascade and its regulation in plants responding to cold stress. Int J Mol Sci. 2022;23:1549 35163471 10.3390/ijms23031549 PMC8835792

[12] Knight MR, Knight H. Low-temperature perception leading to gene expression and cold tolerance in higher plants. New Phytol. 2012;195:737–51 22816520 10.1111/j.1469-8137.2012.04239.x

[13] Usadel B, BlÄSing OE, Gibon Y. et al. Multilevel genomic analysis of the response of transcripts, enzyme activities and metabolites in *Arabidopsis* rosettes to a progressive decrease of temperature in the non-freezing range. Plant Cell Environ. 2008;31:518–47 18088337 10.1111/j.1365-3040.2007.01763.x

[14] Janská A, Marsík P, Zelenková S. et al. Cold stress and acclimation – what is important for metabolic adjustment? Plant Biol (Stuttg). 2010;12:395–405 20522175 10.1111/j.1438-8677.2009.00299.x

[15] Alcázar R, Cuevas JC, Planas J. et al. Integration of polyamines in the cold acclimation response. Plant Sci. 2011;180:31–8 21421344 10.1016/j.plantsci.2010.07.022

[16] Raza A, Charagh S, Najafi-Kakavand S. et al. Role of phytohormones in regulating cold stress tolerance: physiological and molecular approaches for developing cold-smart crop plants. Plant Stress. 2023;8:100152

[17] Raza A, Tabassum J, Kudapa H. et al. Can omics deliver temperature resilient ready-to-grow crops? Crit Rev Biotechnol. 2021;41:1209–32 33827346 10.1080/07388551.2021.1898332

[18] Zhao Y, Zhou M, Xu K. et al. Integrated transcriptomics and metabolomics analyses provide insights into cold stress response in wheat. Crop Journal. 2019;7:857–66

[19] Jin J, Zhang H, Zhang J. et al. Integrated transcriptomics and metabolomics analysis to characterize cold stress responses in *Nicotiana tabacum*. BMC Genomics. 2017;18:496 28662642 10.1186/s12864-017-3871-7 PMC5492280

[20] Wang X, Liu Y, Han Z. et al. Integrated transcriptomics and metabolomics analysis reveal key metabolism pathways contributing to cold tolerance in peanut. Front Plant Sci. 2021;12:752474 34899780 10.3389/fpls.2021.752474 PMC8652294

[21] Zheng L, Wu W, Gao Y. et al. Integrated transcriptome, small RNA and degradome analysis provide insights into the transcriptional regulatory networks underlying cold acclimation in jojoba. Sci Hortic. 2022;299:111050

[22] Moon H-I, Lee J, Zee OP. et al. The effect of flavonol glycoside on the expressions of matrix metalloproteinase-1 in ultraviolet-irradiated cultured human skin fibroblasts. J Ethnopharmacol. 2005;101:176–9 16009522 10.1016/j.jep.2005.04.032

[23] Wang Y-N, Tang L, Hou Y. et al. Differential transcriptome analysis of leaves of tea plant (*Camellia sinensis*) provides comprehensive insights into the defense responses to Ectropis oblique attack using RNA-Seq. Funct Integr Genomics. 2016;16:383–98 27098524 10.1007/s10142-016-0491-2

[24] Gautam H, Sharma A, Trivedi PK. The role of flavonols in insect resistance and stress response. Curr Opin Plant Biol. 2023;73:102353 37001187 10.1016/j.pbi.2023.102353

[25] Herrmann K . Flavonols and flavones in food plants: a review. Int J Food Sci Technol. 1976;11:433–48

[26] Shah A, Smith DL. Flavonoids in agriculture: chemistry and roles in, biotic and abiotic stress responses, and microbial associations. Agronomy. 2020;10:1209

[27] Winkel-Shirley B . Flavonoid biosynthesis. A colorful model for genetics, biochemistry, cell biology, and biotechnology. Plant Physiol. 2001;126:485–93 11402179 10.1104/pp.126.2.485 PMC1540115

[28] Li S . Transcriptional control of flavonoid biosynthesis. Plant Signal Behav. 2014;9:e27522 24393776 10.4161/psb.27522 PMC4091223

[29] Zhang X, Su N, Jia L. et al. Transcriptome analysis of radish sprouts hypocotyls reveals the regulatory role of hydrogen-rich water in anthocyanin biosynthesis under UV-A. BMC Plant Biol. 2018;18:227 30305047 10.1186/s12870-018-1449-4 PMC6180623

[30] Stracke R, Ishihara H, Huep G. et al. Differential regulation of closely related R2R3-MYB transcription factors controls flavonol accumulation in different parts of the *Arabidopsis thaliana* seedling. Plant J. 2007;50:660–77 17419845 10.1111/j.1365-313X.2007.03078.x PMC1976380

[31] Mehrtens F, Kranz H, Bednarek P. et al. The Arabidopsis transcription factor MYB12 is a flavonol-specific regulator of phenylpropanoid biosynthesis. Plant Physiol. 2005;138:1083–96 15923334 10.1104/pp.104.058032 PMC1150422

[32] Luo J, Butelli E, Hill L. et al. AtMYB12 regulates caffeoyl quinic acid and flavonol synthesis in tomato: expression in fruit results in very high levels of both types of polyphenol. Plant J. 2008;56:316–26 18643978 10.1111/j.1365-313X.2008.03597.x

[33] Qi T, Song S, Ren Q. et al. The jasmonate-ZIM-domain proteins interact with the WD-repeat/bHLH/MYB complexes to regulate jasmonate-mediated anthocyanin accumulation and trichome initiation in *Arabidopsis thaliana*. Plant Cell. 2011;23:1795–814 21551388 10.1105/tpc.111.083261 PMC3123955

[34] Tamari G, Borochov A, Atzorn R. et al. Methyl jasmonate induces pigmentation and flavonoid gene expression in petunia corollas: a possible role in wound response. Physiol Plant. 1995;94:45–50

[35] Hu Y, Jiang Y, Han X. et al. Jasmonate regulates leaf senescence and tolerance to cold stress: crosstalk with other phytohormones. J Exp Bot. 2017;68:1361–9 28201612 10.1093/jxb/erx004

[36] Turner JG, Ellis C, Devoto A. The jasmonate signal pathway. Plant Cell. 2002;14:S153–64 12045275 10.1105/tpc.000679 PMC151253

[37] Wasternack C, Hause B. Jasmonates: biosynthesis, perception, signal transduction and action in plant stress response, growth and development. An update to the 2007 review in Annals of Botany. Ann Bot. 2013;111:1021–58 23558912 10.1093/aob/mct067 PMC3662512

[38] Chini A, Fonseca S, Fernández G. et al. The JAZ family of repressors is the missing link in jasmonate signalling. Nature. 2007;448:666–71 17637675 10.1038/nature06006

[39] Thines B, Katsir L, Melotto M. et al. JAZ repressor proteins are targets of the SCF(COI1) complex during jasmonate signalling. Nature. 2007;448:661–5 17637677 10.1038/nature05960

[40] Du H, Liu H, Xiong L. Endogenous auxin and jasmonic acid levels are differentially modulated by abiotic stresses in rice. Front Plant Sci. 2013;4:397 24130566 10.3389/fpls.2013.00397 PMC3793129

[41] Ding F, Wang C, Xu N. et al. Jasmonic acid-regulated putrescine biosynthesis attenuates cold-induced oxidative stress in tomato plants. Sci Hortic. 2021;288:110373

[42] Habibi F, Ramezanian A, Rahemi M. et al. Postharvest treatments with γ-aminobutyric acid, methyl jasmonate, or methyl salicylate enhance chilling tolerance of blood orange fruit at prolonged cold storage. J Sci Food Agric. 2019;99:6408–17 31283020 10.1002/jsfa.9920

[43] Zhao M-L, Wang J-N, Shan W. et al. Induction of jasmonate signalling regulators MaMYC2s and their physical interactions with MaICE1 in methyl jasmonate‐induced chilling tolerance in banana fruit. Plant Cell Environ. 2013;36:30–51 22651394 10.1111/j.1365-3040.2012.02551.x

[44] Ding F, Wang C, Zhang S. et al. A jasmonate-responsive glutathione S-transferase gene *SlGSTU24* mitigates cold-induced oxidative stress in tomato plants. Sci Hortic. 2022;303:111231

[45] Hu Y, Jiang L, Wang F. et al. Jasmonate regulates the inducer of cbf expression-C-repeat binding factor/DRE binding factor1 cascade and freezing tolerance in *Arabidopsis*. Plant Cell. 2013;25:2907–24 23933884 10.1105/tpc.113.112631 PMC3784588

[46] Sturtevant D, Lu S, Zhou ZW. et al. The genome of jojoba (*Simmondsia chinensis*): a taxonomically isolated species that directs wax ester accumulation in its seeds. Sci Adv. 2020;6:eaay3240 32195345 10.1126/sciadv.aay3240 PMC7065883

[47] Al-Dossary O, Alsubaie B, Kharabian-Masouleh A. et al. The jojoba genome reveals wide divergence of the sex chromosomes in a dioecious plant. Plant J. 2021;108:1283–94 34570389 10.1111/tpj.15509 PMC9293028

[48] Xu W, Dubos C, Lepiniec L. Transcriptional control of flavonoid biosynthesis by MYB-bHLH-WDR complexes. Trends Plant Sci. 2015;20:176–85 25577424 10.1016/j.tplants.2014.12.001

[49] Abubakar AS, Feng X, Gao G. et al. Genome wide characterization of R2R3 MYB transcription factor from *Apocynum venetum* revealed potential stress tolerance and flavonoid biosynthesis genes. Genomics. 2022;114:110275 35108591 10.1016/j.ygeno.2022.110275

[50] Wang N, Xu H, Jiang S. et al. MYB12 and MYB22 play essential roles in proanthocyanidin and flavonol synthesis in red-fleshed apple (*Malus sieversii* f. *niedzwetzkyana*). Plant J. 2017;90:276–92 28107780 10.1111/tpj.13487

[51] Wang M, Ren T, Huang R. et al. Overexpression of an *Apocynum venetum* flavonols synthetase gene confers salinity stress tolerance to transgenic tobacco plants. Plant Physiol Biochem. 2021;162:667–76 33780740 10.1016/j.plaphy.2021.03.034

[52] Gharibi S, Tabatabaei BE, Saeidi G. et al. Effect of drought stress on total phenolic, lipid peroxidation, and antioxidant activity of *Achillea* species. Appl Biochem Biotechnol. 2016;178:796–809 26541161 10.1007/s12010-015-1909-3

[53] He J, Yao L, Pecoraro L. et al. Cold stress regulates accumulation of flavonoids and terpenoids in plants by phytohormone, transcription process, functional enzyme, and epigenetics. Crit Rev Biotechnol. 2022;18:1–18 10.1080/07388551.2022.2053056 35848841

[54] Schulz E, Tohge T, Zuther E. et al. Flavonoids are determinants of freezing tolerance and cold acclimation in *Arabidopsis thaliana*. Sci Rep. 2016;6:34027 27658445 10.1038/srep34027 PMC5034326

[55] Wang L, Tu Y, Lian T. et al. Distinctive antioxidant and antiinflammatory effects of flavonols. J Agric Food Chem. 2006;54:9798–804 17177504 10.1021/jf0620719

[56] Aderogba MA, McGaw LJ, Ogundaini AO. et al. Antioxidant activity and cytotoxicity study of the flavonol glycosides from *Bauhinia galpinii*. Nat Prod Res. 2007;21:591–9 17613816 10.1080/14786410701369557

[57] Kim GN, Jang HD. Flavonol content in the water extract of the mulberry (*Morus alba* L.) leaf and their antioxidant capacities. J Food Sci. 2011;76:C869–73 22417484 10.1111/j.1750-3841.2011.02262.x

[58] Ruan J, Zhou Y, Zhou M. et al. Jasmonic acid signaling pathway in plants. Int J Mol Sci. 2019;20:2479 31137463 10.3390/ijms20102479 PMC6566436

[59] Hewedy OA, Elsheery NI, Karkour AM. et al. Jasmonic acid regulates plant development and orchestrates stress response during tough times. Environ Exp Bot. 2023;208:105260

[60] Garoosi MK, Sanjarian F, Chaichi M. The role of γ-aminobutyric acid and salicylic acid in heat stress tolerance under salinity conditions in *Origanum vulgare* L. PLoS One. 2023;18:e0288169 37418380 10.1371/journal.pone.0288169 PMC10328350

[61] Wang Z, Wong DCJ, Wang Y. et al. GRAS-domain transcription factor PAT1 regulates jasmonic acid biosynthesis in grape cold stress response. Plant Physiol. 2021;186:1660–78 33752238 10.1093/plphys/kiab142 PMC8260143

[62] Ding F, Ren L, Xie F. et al. Jasmonate and melatonin act synergistically to potentiate cold tolerance in tomato plants. Front Plant Sci. 2021;12:763284 35069620 10.3389/fpls.2021.763284 PMC8776829

[63] Naik J, Misra P, Trivedi PK. et al. Molecular components associated with the regulation of flavonoid biosynthesis. Plant Sci. 2022;317:111196 35193745 10.1016/j.plantsci.2022.111196

[64] Du T, Fan Y, Cao H. et al. Transcriptome analysis revealed key genes involved in flavonoid metabolism in response to jasmonic acid in pigeon pea (*Cajanus cajan* (L.) Millsp.). Plant Physiol Biochem. 2021;168:410–22 34715566 10.1016/j.plaphy.2021.10.022

[65] Król P, Igielski R, Pollmann S. et al. Priming of seeds with methyl jasmonate induced resistance to hemi-biotroph *Fusarium oxysporum* f.sp. *lycopersici* in tomato via 12-oxo-phytodienoic acid, salicylic acid, and flavonol accumulation. J Plant Physiol. 2015;179:122–32 25867625 10.1016/j.jplph.2015.01.018

[66] Thiruvengadam M, Rekha K, Rajakumar G. et al. Enhanced production of anthraquinones and phenolic compounds and biological activities in the cell suspension cultures of *Polygonum multiflorum*. Int J Mol Sci. 2016;17:1912 27854330 10.3390/ijms17111912 PMC5133909

[67] Santner A, Estelle M. The JAZ proteins link jasmonate perception with transcriptional changes. Plant Cell. 2008;19:3839–42 10.1105/tpc.107.056960 PMC2217635 18165326

[68] Wager A, Browse J. Social network: JAZ protein interactions expand our knowledge of jasmonate signaling. Front Plant Sci. 2012;3:41 22629274 10.3389/fpls.2012.00041 PMC3355530

[69] Xie Y, Tan H, Ma Z. et al. DELLA proteins promote anthocyanin biosynthesis via sequestering MYBL2 and JAZ suppressors of the MYB/bHLH/WD40 complex in *Arabidopsis thaliana*. Mol Plant. 2016;9:711–21 26854848 10.1016/j.molp.2016.01.014

[70] Zhang X, Li L, He Y. et al. The CsHSFA-CsJAZ6 module-mediated high temperature regulates flavonoid metabolism in *Camellia sinensis*. Plant Cell Environ. 2023;46:2401–18 37190917 10.1111/pce.14610

[71] Huang X, Wu Y, Zhang S. et al. Overexpression of *RuFLS2* enhances flavonol-related substance contents and gene expression levels. Int J Mol Sci. 2022;23:14230 36430708 10.3390/ijms232214230 PMC9699159

[72] Li J, Luan Q, Han J. et al. CsMYB60 directly and indirectly activates structural genes to promote the biosynthesis of flavonols and proanthocyanidins in cucumber. Hortic Res. 2020;7:103 32637131 10.1038/s41438-020-0327-z PMC7327083

[73] Zhai R, Zhao Y, Wu M. et al. The MYB transcription factor PbMYB12b positively regulates flavonol biosynthesis in pear fruit. BMC Plant Biol. 2019;19:85 30791875 10.1186/s12870-019-1687-0 PMC6385385

[74] Tohge T, Nishiyama Y, Hirai MY. et al. Functional genomics by integrated analysis of metabolome and transcriptome of Arabidopsis plants over-expressing an MYB transcription factor. Plant J. 2005;42:218–35 15807784 10.1111/j.1365-313X.2005.02371.x

[75] Liu W, Feng Y, Yu S. et al. The flavonoid biosynthesis network in plants. Int J Mol Sci. 2021;22:12824 34884627 10.3390/ijms222312824 PMC8657439

[76] Li S, Zachgo S. TCP3 interacts with R2R3-MYB proteins, promotes flavonoid biosynthesis and negatively regulates the auxin response in *Arabidopsis thaliana*. Plant J. 2013;76:901–13 24118612 10.1111/tpj.12348

[77] An J-P, Wang X-F, Zhang X-W. et al. Apple B-box protein BBX37 regulates jasmonic acid mediated cold tolerance through the JAZ-BBX37-ICE1-CBF pathway and undergoes MIEL1-mediated ubiquitination and degradation. New Phytol. 2021;229:2707–29 33119890 10.1111/nph.17050

[78] Ding F, Wang X, Li Z. et al. Jasmonate positively regulates cold tolerance by promoting ABA biosynthesis in tomato. Plants (Basel). 2022;12:60 36616188 10.3390/plants12010060 PMC9823970

[79] Geng H, Shi L, Li W. et al. Gene expression of jojoba (*Simmondsia chinensis*) leaves exposed to drying. Environ Exp Bot. 2008;63:137–46

[80] Chong J, Yamamoto M, Xia J. MetaboAnalystR 2.0: from raw spectra to biological insights. Meta. 2019;9:57 10.3390/metabo9030057 PMC6468840 30909447

[81] Rodríguez-Vargas S, Sánchez-García A, Martínez-Rivas Jose M. et al. Fluidization of membrane lipids enhances the tolerance of *Saccharomyces cerevisiae* to freezing and salt stress. Appl Environ Microbiol. 2007;73:110–6 17071783 10.1128/AEM.01360-06 PMC1797130

[82] Melcarek PK, Brown GN. Effects of chill stress on prompt and delayed chlorophyll fluorescence from leaves. Plant Physiol. 1977;60:822–5 16660193 10.1104/pp.60.6.822 PMC542726

[83] Gould PD, Diaz P, Hogben C. et al. Delayed fluorescence as a universal tool for the measurement of circadian rhythms in higher plants. Plant J. 2009;58:893–901 19638147 10.1111/j.1365-313X.2009.03819.x

[84] Zhang W, Tang Y, Hu Y. et al. Arabidopsis NF-YCs play dual roles in repressing brassinosteroid biosynthesis and signaling during light-regulated hypocotyl elongation. Plant Cell. 2021;33:2360–74 33871651 10.1093/plcell/koab112 PMC8364247

[85] Sun X, Wang P, Jia X. et al. Improvement of drought tolerance by overexpressing *MdATG18a* is mediated by modified antioxidant system and activated autophagy in transgenic apple. Plant Biotechnol J. 2018;16:545–57 28703378 10.1111/pbi.12794 PMC5787838

[86] Zhou Z, Bi G, Zhou J-M. Luciferase complementation assay for protein‐protein interactions in plants. Curr Protoc Plant Biol. 2018;3:42–50 30040251 10.1002/cppb.20066

[87] Cheng M-C, Enderle B, Kathare PK. et al. PCH1 and PCHL directly interact with PIF1, promote its degradation, and inhibit its transcriptional function during photomorphogenesis. Mol Plant. 2020;13:499–514 32061894 10.1016/j.molp.2020.02.003 PMC7167218

[88] Cao X, Xie H, Song M. et al. Cut–dip–budding delivery system enables genetic modifications in plants without tissue culture. Innovation. 2023;4:100345 36387605 10.1016/j.xinn.2022.100345 PMC9661722

[89] Schmülling T, Schell J, Spena A. Single genes from *Agrobacterium rhizogenes* influence plant development. EMBO J. 1988;7:2621–9 15977331 10.1002/j.1460-2075.1988.tb03114.x PMC457048

